# Protocol for the "four steps to control your fatigue (4-STEPS)" randomised controlled trial: a self-regulation based physical activity intervention for patients with unexplained chronic fatigue

**DOI:** 10.1186/1471-2458-12-202

**Published:** 2012-03-19

**Authors:** Marta Marques, Véronique De Gucht, Stan Maes, Isabel Leal

**Affiliations:** 1 Health Psychology Department, Leiden university, Wassenaarseweg 52, P.O. BOX 955, 2300 RB Leiden, The Netherlands; 2 Research Unit on Psychology and Health (UIPES), ISPA- University Institute, Rua Jardim do Tabaco, 34, 1149-041 Lisboa, Portugal

**Keywords:** Unexplained Chronic Fatigue, Physical activity, Self-Regulation, Randomized Controlled Trial

## Abstract

**Background:**

Unexplained Chronic Fatigue is a medical condition characterized by the presence of persistent, severe and debilitating medically unexplained fatigue, leading to impaired functioning and lower quality of life. Research suggests that physical activity can contribute to the reduction of fatigue and other somatic symptoms and can thus significantly improve physical functioning and quality of life in these patients. Based on the self-regulation (SR) theory of behaviour change, we developed a brief physical activity program for patients suffering from unexplained chronic fatigue which focuses on the training of self-regulation skills, the "4-STEPS to control your fatigue" program.

**Methods/Design:**

This is a multi-centre, randomised controlled trial (RCT) that will be carried out in local primary care centres and at the Portuguese Fibromyalgia and Chronic Fatigue Syndrome Patients Association. Patients aged between 18 and 65 and fulfilling operationalized criteria for Idiopathic Chronic Fatigue (ICF) and Chronic Fatigue Syndrome (CFS) will be recruited and randomly allocated to standard care (SC) or standard care plus a self-regulation based physical activity program (4-STEPS). Patients will be assessed at baseline, after the intervention (3 months) and at 12 months follow-up. The primary outcome is fatigue severity.

**Discussion:**

The results of the RCT will provide information about the effectiveness of a brief self-regulation intervention for promoting physical activity in patients with unexplained chronic fatigue. If the program proves to be effective, it may be considered as an adjunctive treatment for these patients.

**Trial Registration:**

ISRCTN: ISRCTN70763996

## Background

Fatigue is a common symptom in adults worldwide, being reported by around 20% of patients seeking medical care [1]. A recent epidemiological review concluded that physical activity can reduce fatigue and improve energy [1]. Recent literature emphasizes that fatigue should be considered as a multidimensional concept, incorporating both physical and mental fatigue [2]. Due to its subjective nature, fatigue is difficult to define and measure. It is a personal experience that cannot be objectively measured [3].

In most cases, fatigue is transient, but for some, fatigue symptoms become chronic, resulting in disability [4]. When fatigue lasts for at least six months, is not alleviated by rest, is debilitating, results in a significant reduction of daily activities and cannot be explained by an organic disease (unexplained chronic fatigue), it is classified as Idiopathic Chronic Fatigue (ICF) [4,5]. If ICF is accompanied by four or more of the following symptoms - unrefreshing sleep, lengthy malaise after exertion (lasting for over 24 hours), impaired memory or concentration, sore throat, tender cervical or axillary lymph nodes, muscle pain, multi-joint pain without swelling or redness and headaches of a new type or severity - it is diagnosed as Chronic Fatigue Syndrome (CFS) according to the Center for Disease Control (CDC) criteria [6].

CFS prevalence has been reported to be in between 0.2% and 2% in general population samples [7]. Prevalence rates vary according to several factors such as the criteria used to diagnose CFS [4]. In terms of prognosis, a systematic review conducted by Cairns and Hotpof [8] found that full recovery from untreated CFS is rare. It is more common for patients to experience an improvement in symptom severity. CFS etiology remains unknown [4] and it is considered to be associated with a combination of several predisposing (e.g. genes), precipitating (e.g. life events) and perpetuating (e.g. physical inactivity) factors [9].

Patients with unexplained chronic fatigue often report complaints related to exercise intolerance and post-exertional malaise [4]. Prins, van der Meer and Bleijenberg [9] considered patients' perceptions and expectations related to symptom exacerbation as a consequence of exercise, to be the main cause of the reduced levels of physical activity found in patients with unexplained chronic fatigue, rather than physical fitness limitations per se. Other studies pointed out that lack of physical activity and excessive resting are factors that result in physical deconditioning which, in turn, perpetuates fatigue and physical disability [10]. It has therefore been recommended that ICF/CFS patients engage in physical activity instead of refraining from it to manage their symptoms [10-12].

To promote physical activity in ICF/CFS, Graded Exercise Therapy (GET) has been recommended. GET is based on the assumption that in patients with unexplained chronic fatigue, physical activity must be initiated at a level that doesn't exacerbate symptoms and must be gradually increased until patients reach an optimal level of activity. Graded exercise programs follow the exercise prescription guidelines from the American College of Sports Medicine [13], tailored to each patient's level of physical activity. GET is usually delivered by an exercise physiologist or physical therapist, and consists of supervised exercise sessions and/or home-based exercise prescription (e.g. walking). Research has demonstrated that GET has a positive effect on physical activity and leads to decreased levels of fatigue [14-17].

Cognitive-behavioral therapy (CBT) has also demonstrated to be an effective treatment approach to CF. CBT interventions developed for ICF/CFS patients focus primarily on changing illness cognitions and expectations, as well as increasing control over symptoms. The CBT model makes a distinction between precipitating factors - factors that contribute to the initiation of a health problem - and perpetuating factors - factors that contribute to the maintenance of the health problem [18]. Since physical inactivity is considered to be a perpetuating factor of unexplained chronic fatigue, most CBT interventions also focus on physical activity promotion. A recent review conducted by Price and colleagues [19] concluded that CBT interventions have a positive effect on fatigue, depression and anxiety in ICF/CFS patients.

In patients with unexplained chronic fatigue a "boom and bust pattern" is frequently found, that is, a systematic alternation between periods of excessive activity (when feeling good), and, as a consequence of that, feeling extremely fatigued and having to rest for longer periods of time [20,21]. Pacing, which is having an appropriate balance between activity and rest, is considered to be an important technique for reducing fatigue symptoms. Pacing implies that patients are encouraged to set realistic goals on a daily basis in terms of activity and rest periods; it is often combined with graded exercise [22,23].

CBT uses several self-regulation (SR) techniques. SR can be defined as a sequence of actions and/or steering processes intended to attain a personal goal [24]. According to SR theory, individuals set personally important goals that guide their behavior [25]. In this goal-guidance process, self-regulation cognitions (e.g. self-efficacy expectations and autonomous/controlled motivation), emotions (positive and negative affect) and skills (e.g. self-monitoring and feedback), are considered to play an important role, both in goal setting, active goal pursuit and goal maintenance/attainment [25]. As a consequence, SR models not only contribute to our understanding of the influence of life goals on medically unexplained physical symptoms (MUPS) in general, and fatigue in particular [26], but interventions based upon these models may also encourage patients to change their personal goals from symptom avoidance to more active and positive goals [27]. In their review, Maes & Karoly [25] distinguished a number of self-regulation strategies associated with behavior change and derived a set of guidelines for interventions. These self-regulation core processes are: realistic outcome expectations; illness representations; goal setting (personal goals, ownership); planning; progress evaluation and feedback; efficacy support; attention and emotion control; control over competing goals; self-monitoring; self-reinforcement; facilitate social support; goal reformulation; relapse prevention; anticipatory coping [25].

One of the main intervention techniques using self-regulation principles is Motivational Interviewing (MI), developed by Miller & Rollnick [28]. MI is a directive, client-centered technique for eliciting behavior change, by helping clients to explore and resolve perceived conflicts (ambivalence) with respect to behavior change and analyze the discrepancies between their current behavior and their life goals and values. It aims at increasing autonomous regulation and self-efficacy (i.e. confidence in one's ability to perform a certain behavior) and promotes the transition from planning (motivational phase) to action (goal pursuit). MI has been successful in promoting physical activity in healthy populations [29], but there is limited research on MI interventions in patients with ICF/CFS and other MUPS. Powell and colleagues [30] evaluated an intervention based upon MI techniques to encourage graded physical exercise in CFS patients. The intervention led to increased physical functioning and decreased fatigue. In addition, the minimum intervention condition, consisting of only two face-to-face sessions, proved to be as successful as more extended versions of the program. A study conducted with fibromyalgia patients also showed a significant increase in physical exercise frequency, as well as reduced pain and physical impairment after an MI intervention [31].

Based on the empirical literature we developed a brief self-regulation based physical activity program for patients suffering from unexplained chronic fatigue, the "4-Steps to control your fatigue" (4-STEPS) program. In this intervention program, the combination of graded exercise and pacing is incorporated [22]. The development and evaluation of brief interventions is important because of the time, money and energy consuming efforts associated with longer interventions.

The main objective of the study is to evaluate the efficacy of the 4-STEPS program in promoting physical activity and in reducing fatigue.

## Methods/Design

### Design of the study

**Figure 1:**
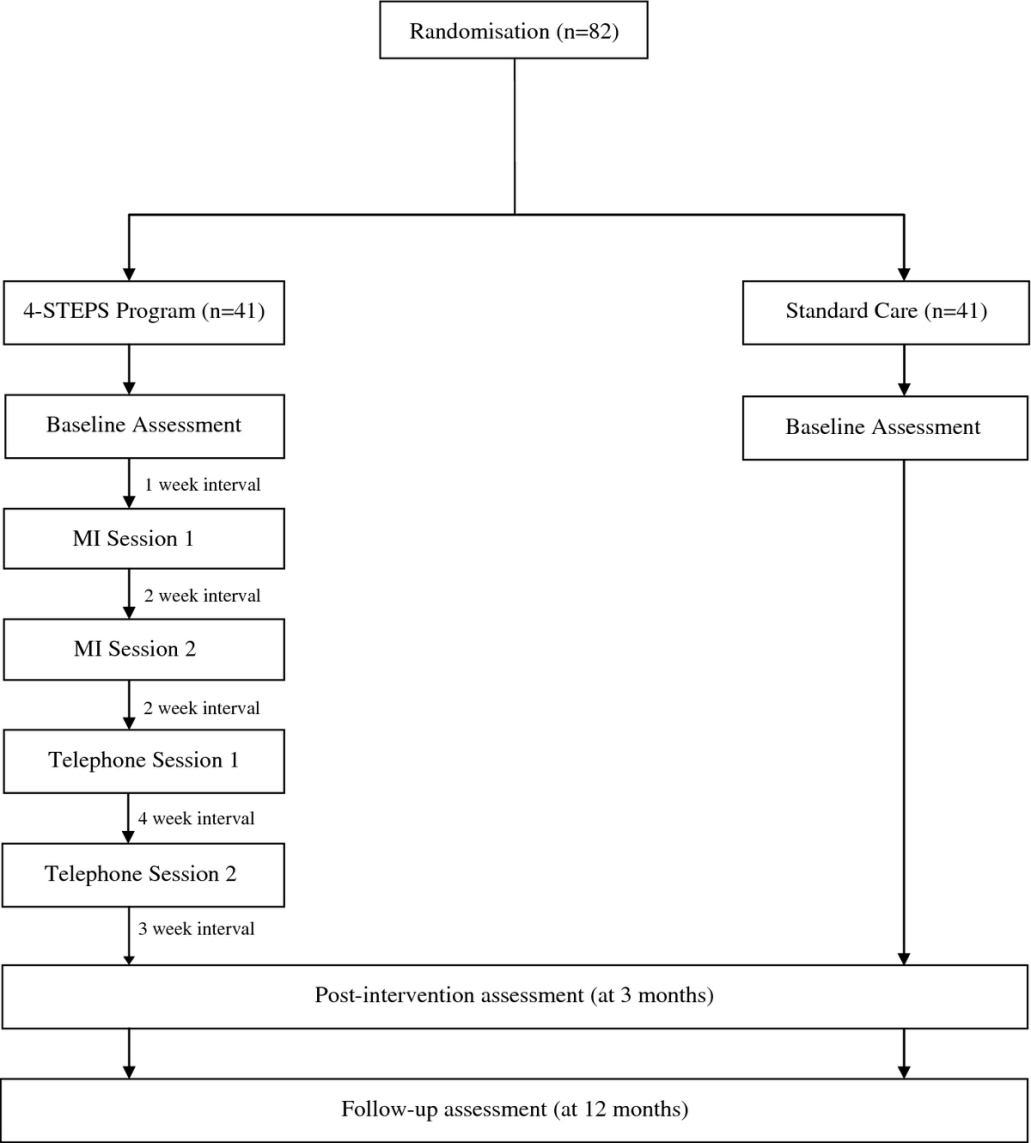
**Recruitment and design flow diagram**.

This is a two-arm multi-centre randomised controlled trial in patients who meet operationalised criteria for either ICF or CFS. It consists of a 3-month intervention and a 12-month follow-up phase (Figure 1). There are 3 measurement points: baseline, post-test (3 months) and follow-up at 12 months.

Approval for the trial was obtained from the Ethics Committee of the North Regional Health Administration (Ref: 27.09), from the directors of each participating health care institution and from the Portuguese Fibromyalgia and Chronic Fatigue Syndrome Patient Association.

### Setting

The trial, including measurements and intervention sessions is conducted in five Portuguese primary care centres, in a private practice clinic and at the Portuguese Fibromyalgia and Chronic Fatigue Syndrome Patient Association.

### Participants

Patients attending their physician with a main complaint of unexplained fatigue of at least six months duration are recruited for the study. Inclusion criteria are: meeting the operationalised criteria for ICF or CFS (CDC criteria) [6]; aged between 18 and 65; fluent in spoken Portuguese; capacity to provide informed consent. Exclusion criteria are: presence of a concurrent somatic condition which can explain the fatigue symptoms; patients with severe psychiatric disorders.

### Power calculation

The sample size was calculated based on the primary outcome (fatigue severity). A power calculation [32], using an independent samples t-test (5% level of significance) indicated that a sample size of 34 will have 80% power to detect a mean difference of 7 points [33,34] between the intervention and the control group, on the subjective experience of fatigue subscale of the Checklist of Individual Strength (CIS-R) [35].

Anticipating a drop-out rate of 20% [33], we aim at recruiting 41 subjects per group at baseline.

### Randomization method

Participants are recruited from consecutive referrals and allocated to one of the two trial arms by the research team leader. Randomisation sequence is stratified by centre with a 1:1 allocation to the two treatment arms. Standard care is given to all participants and the intervention group additionally receives the 4-STEPS program.

### Enrolment procedure

Different procedures are followed for recruiting patients from the primary health care institutions on the one hand and from the patient association on the other.

#### Health care institutions

First, patients that meet the inclusion/exclusion criteria are approached for participation by their primary care physician. Secondly, the physician provides a brief explanation of the study to the patient and asks for the patient's authorization to be contacted by a member of the research team. Third, patients receive information from the principal investigator about the trial and what their participation involves. Finally, patients are assigned to either the intervention or the control condition by randomized sampling. All patients willing to participate sign a written informed consent before being enrolled in the study.

#### National fibromyalgia and chronic fatigue patient association

Initially, a letter containing information about the trial, what participation involves and the written informed consent form is sent to all members of the association from the Lisbon and Porto region that meet the inclusion/exclusion criteria and have previously indicated they were available for participation in research studies. Patients who wish to participate in the study return the written informed consent and are subsequently randomly assigned to either the intervention or the control group.

### Interventions

#### Control group

In addition to standard medical care, patients that are assigned to this group receive a flyer with information about the general health benefits of physical activity and the current physical activity guidelines for adults [13].

#### Intervention group

In addition to standard care, patients in the intervention group receive the 4-STEPS program that consists of:

1. Two face-to-face individual motivational interviewing (MI) sessions aimed at exploring important health and life goals, increasing participants' motivation and confidence to be physically active and setting a specific personal physical activity goal. The first MI session takes place 1 week after the baseline assessment and the second MI session takes place 2 weeks after the first. The MI session is delivered by a psychologist with MI training (member of the research team). The duration of the sessions is approximately 1 hour. Details on the topics addressed in the MI sessions are presented in Table 1.

2. Two brief telephone counseling sessions: These sessions take about 20 minutes and are provided 2 weeks and 6 weeks after the last MI session. Details on the topics addressed during the telephone sessions are presented in Table 1.

**Table 1:** 4-STEPS sessions content

4-STEPS sessions	Topics
**Motivational Interviewing- Session 1**	✓ Listen to patient complaints, with emphasis on fatigue.
	✓ Illness perceptions.
	✓ Exploration of patients' present physical activity behavior.
	✓ Current health behaviors and other activities are explored (physical activity, sleep, pain, etc.).
	✓ Discussion of the information on the booklet given previously (specially the relation between CF major symptoms and physical activity behavior and boom-bust pattern).
	✓ Motivation and Confidence Scales: Exploration of the willingness to engage in physical activity (or adjust current physical activity behavior) and the confidence in one's own capability to achieve this behavior with success.
	✓ Discussion of the pros and cons (not-) to change physical activity behavior (Decisional Balance).
	✓ Social support network is addressed.

**Motivational Interviewing- Session 2**	✓ Exploration of possible competing goals with physical activity (e.g. housework chores) and link to core values.
	✓ Strategies to overcome obstacles to physical activity and competing goals are addressed.
	✓ Based on the register of daily activities and steps taken (pedometer register) and GET scheme for physical activity, the patient establishes a personal physical activity goal for the following two months.
	✓ Discuss the possible reasons for perpetuation of symptoms and difficulty to change health behaviors in CF patients, namely perfectionism, fear of movement, presence of stressful situations and boom-bust pattern. (some are already mentioned at the first motivational interview and are now emphasized).
	✓ Exploration of emotion control importance and techniques (e.g. relaxation techniques).

**Telephone sessions:**	✓ Revision of the physical activity goal planning (adequacy to the patients' present situation).
	✓ Relapse prevention (strategies to overcome new or persisting difficulties to physical activity and emotion control strategies).

3. Self-regulation (SR) booklets: There are two booklets that are designed to help patients change their level of physical activity (Informational booklet and Workbook). The Informational booklet is provided at the end of the baseline assessment, the "Step 1" part of the Workbook is provided at the first MI session and the parts "Step 2", "Step 3" and "Step 4" are given during the second MI session. Details on the topics that are addressed in the SR booklets are presented in Table 2.

4. A pedometer to register physical activity on a daily basis (steps taken) during the 3 month intervention period. Instructions on how to use the pedometer are given in the baseline assessment session (Table 2).

5. Daily activities record (Table 2): Patients receive several daily activity records (physical activities, mental activities and rest). The first daily activity record is given to the patient at the end of the first MI session; patients are asked to fill out the activity record in the time period between the first and second MI session. This homework assignment aims at evaluating the patients' daily activities management and possibly recognizing an erratic pattern of rest and activity (boom and bust cycle). At the end of the second MI session, patients receive daily activities records that can be used to monitor changes in daily activity patterns during the subsequent nine weeks.

6. Leaflet for family (Table 2): At the end of the first MI session patients receive a leaflet for their partner or significant other in order to increase social support.

**Table 2:** 4-STEPS Materials content

4-STEPS materials	TOPICS
**Self-regulation Informational booklet**	✓ Overview of Chronic Fatigue: CDC diagnosis criteria, epidemiology information, etiology, possible factors that contribute to a better or words prognosis (e.g. physical inactivity/activity).
	✓ Physical activity: current physical activity guidelines for adults in general population, physical and psychological benefits of physical activity, benefits of physical activity for CF. Information about different types of physical activities (that are appropriated for CF) such as walking as well the importance and illustration of relaxation training.
	✓ Link between CF symptoms and physical (in-) activity (deconditioning cycle) and boom-bust pattern (erratic pattern of rest and activity).

**Self-regulation Workbook**	*STEP 1 - "Am I ready to start?"*
	✓ Motivation and Confidence Scales: Patients' willingness and confidence to do adjusted levels of physical activity is assessed on a scale for ranging from 0% (not at all) to 100% (very much).
	✓ Decisional Balance: list of the pros and cons (not-) to change in relation to physical activity.
	✓ Competing goals with exercise: information on the importance o analyzing possible competing goals (or conflicting activities) with exercise that may be refraining patients from physical activity. List of two main activities (e.g. other leisure activities).
	✓ Activity/Rest Diary and Pedometer log - instructions to use for two weeks (interval between MI sessions) and the importance of the pedometer as a motivational strategy.
	*STEP 2 - "My physical activity goal":*
	✓ GET Scheme and daily activity management: information on the importance of increase physical activity level gradually, consider the way patients feel in different days (different levels if patients is feeling good or bad), importance of rest during physical activity, importance of adjusting physical activity levels to the amount of effort spent in that day (balance between activities and rest).
	✓ Goal setting activity: explanation of how to set a goal (SMART - specific, measurable, achievable, realistic and timely), formulation of a personal physical activity goal to achieve in 2 months time (e.g. to do brisk walking four days per week for 30 minutes (plus 10 minutes break), implementation intentions (type of activity, frequency and intensity, period of the day, with whom, where) and a goal ladder (patients formulate goals for every 2 weeks until reaching the two months time and main goal.
	✓ Information on self-rewarding for each step goal achieved.
	✓ Tips for managing daily activities and incorporate physical activity on the daily routine
	*STEP3 - "Overcoming obstacles":*
	✓ Problem solving activity: List two main physical activity barriers and a strategy to overcome it.
	*STEP 4 - "I am physically active...and I want to keep that way":*
	✓ Relapse prevention information and strategies
	*Resources and support for CF and physical activity*.

**Pedometer Record**	✓ Similar to the record used for the assessment (day of the week and number of steps for each day).
**Daily activities record**	✓ Record containing the days of the week and hours each day. Patients fill the activities they do in each hour of day. The options are: physical activities, other activities (activities requiring mental effort) and rest. Each option is explained in detail.

**Leaflet for family**	✓ Facts about CF.
	✓ Brief information on the relation between physical activity and CF.
	✓ Tips for partners or other relatives to support patients with CF.

### Assessments

Measures will be collected at baseline, 3 months later and 12 months after baseline. All measures are self-report. Data will be collected in both the health care institutions and the patient association, at all times (Table 3). Primary and secondary outcomes, predictors and process evaluation measures are described below. A description of measures and time points can be found in Table 3.

**Table 3:** Measures to be administered at each time point

	Baseline	Post-test	12 months
Demographics	X		
Severe infection	X		
Life event	X		
Medical advice	X		
Duration Symptoms and diagnosis	X		
CFS symptom checklist	X	X	X
Presence of FM symptoms	X	X	X
CIS-20R	X	X	X
Impact of fatigue	X	X	X
NFR	X	X	X
Work related questions	X	X	X
Brief IPQ-R	X	X	X
Brief IPQ-R causes subscale	X		
CERQ positive subscales	X	X	X
PCS	X	X	X
BRIQ	X	X	X
Social support	X	X	X
BSI	X	X	X
Sleep	X	X	X
PHQ-15	X	X	X
SF-12	X	X	X
Physical Activity history	X	X	X
Steps taken (pedometer)	X	X	X
Physical activity intention	X		
Action planning	X		
Coping planning	X		
SRSB Physical activity goal elicitation	X		
SRSB Physical activity goal progress	X	X	X
SRSB goal efficacy scale	X		
SRSB all scales		X	X
Barriers Efficacy	X	X	X
TSRQ	X	X	X
Intervention evaluation (intervention condition)		X	

#### Primary outcome

The primary outcome is the reduction of perceived fatigue severity, which is assessed by means of the Checklist of Individual Strength (CIS-20R) [35]. A difference of 7 points between the intervention and the control group on the main dimension (= the subjective feeling of fatigue subscale) of the CIS-20 R is considered to be a clinically significant difference [33,34].

#### Secondary outcomes

1. Fatigue severity, assessed by means of the CIR-20R [35].

2. ICF and CFS diagnosis will be assessed by means of the CDC criteria and using the CDC-CFS Symptom Inventory [36].

3. Presence of Fibromyalgia symptoms.

4. Fatigue impact is assessed by means of a modified version of the pain interference subscale of the Brief Pain Inventory (BPI) [37].

5. Work or daily activities related fatigue is assessed by means of the Need for Recovery Scale (NFR) [38].

6. Use of health care resources is measured on the basis of two questions: (1) number of visits to the primary care physician and medical specialists, (2) use of medication.

7. Work related information: currently (not) working, if currently working, number of hours working, working part-time due to fatigue, dropped out of work due to fatigue, number of days absent from work.

8. The All-or-nothing and Limiting behaviour scales from The Behaviour Responses to Illness Questionnaire (BRIQ) [39] are used to assed behavioural symptom management.

9. Anxiety and depression are measured with the Brief Symptom Inventory (BSI) [40,41].

10. Quality of sleep is assessed with five questions based on the DSM-IV criteria for sleep disorders [42] and six questions from the Pittsburgh Sleep Quality Index (PSQI) [43].

11. Number and severity of physical symptoms is measured by means of the Patient Health Questionnaire-15 (PHQ-15) [44].

12. Physical and emotional functioning is measured with the Short Form Health Survey-12 (SF-12 V.2) [45].

13. Physical activity: two different measures are used to assess physical activity level. The first is the pedometer (YAMAX SW-200), a portable device that counts the number of steps taken, by detecting hip motions. Participants are asked to use the pedometer on a daily basis for seven consecutive days and register their daily number of steps on a form that is provided to the patient. The second measure is the Sports subscale of the SQUASH [46]; in this subscale participants indicate the type of physical activity they do (e.g. swimming) including the frequency per week (e.g. 3 days per week) and duration per day (e.g. 50 minutes) for each of these activities. The intensity of each of these activities is calculated based on the Ainsworth's Compendium of Physical activities [47].

#### Predictors

1. Demographic information (age, gender and education).

2. Presence of a severe infection prior to the onset of fatigue.

3. Duration of fatigue at baseline.

4. Occurrence and current impact (by means of a Visual Analogue Scale from 0 cm- no impact at all to 10 cm-exterme impact) of a serious life event experienced prior to the onset of fatigue.

5. Patients' illness beliefs are assessed by the Brief Illness Perception Questionnaire (Brief IPQ-R) [48].

6. Three subscales of the Cognitive Emotion Regulation Questionnaire - Short Version (CERQ-short) [49] were used to assess adaptive cognitive coping strategies, and a version of the Pain Catastrophizing Scale (PCS) adapted for fatigue [50] was used to measure catastrophic cognitions related to CF symptoms.

7. Level and range of social support are assessed.

8. Goal progress and self-regulation skills to achieve a personal goal are measured with the Self-regulation Skills Battery (SRSB) [51].

9. Self-efficacy to overcome obstacles to physical activity is assessed with the Barriers Efficacy Scale [52].

10. Autonomous and Coerced motivation to be physically active is assessed using the respective scale from the Treatment Self-regulation Questionnaire (TSRQ) [53].

11. Physical activity intention, action planning and coping planning are assessed using the measures from Sniehotta and colleagues [54].

#### Process evaluation

Participant satisfaction with the self-regulation based 4-steps program will be assessed.

### Analysis plan

We will use the SPSS and AMOS software packages for data analysis. Descriptive analysis will be performed for demographics, clinical information and process evaluation, stratified by treatment condition. Differences between the control and intervention group for the primary and secondary outcome measures will be assessed using MAN(C)OVA and AN(C)OVA. We also intend to use regression analysis and structural equation modelling to analyse longitudinal relationships between predictors, possible mediators and outcomes (path analysis).

## Discussion

Physical activity seems to be very important for patients suffering from unexplained chronic fatigue, while SR interventions seem to be effective in promoting long-term health behaviour change. The 4-STEPS program will be the first brief SR based physical activity intervention for ICF/CFS patients. We are not aware of any RCT to promote physical activity in these patients conducted in Portugal. This RCT will provide information on the efficacy of the intervention as well as on the predictors of physical activity, fatigue and other somatic symptoms. The fact that it is a brief intervention, that requires minimal personal contact with the health care professional and that patients receive self-help materials to support them can also be seen as an advantage from a cost-effectiveness point of view if the trial has a significant effect. If proven effective, this program can be considered as an adjunctive treatment for ICF/CFS.

## Status of the trial

The 4-STEPS RCT started in January 2011 and it is ongoing.

## Abbreviations

CFS: Chronic Fatigue Syndrome; CBT: Cognitive Behavioural Therapy; GET: Graded Exercise Therapy; ICF: Idiopathic Chronic Fatigue; ISRCTN: International Standard Randomised Controlled Trial; MUPS: (Medically Unexplained Physical Symptoms); RCT: Randomised Controlled Trial; SR: Self-regulation; 4-STEPS: "4 Steps to control your fatigue" program.

## Competing interests

The authors declare that they have no competing interests.

## Authors' contributions

MM, VDG and SM contributed to the design of the study and the creation of the Support materials. VDG, SM and IL participate in the study coordination. MM wrote the draft version of the manuscript. All authors reviewed and approved the final manuscript.

## Pre-publication history

The pre-publication history for this paper can be accessed here:

http://www.biomedcentral.com/1471-2458/12/202/prepub
